# Acute Kidney Injury in COVID-19 Pneumonia: A Single-Center Experience in Bahrain

**DOI:** 10.7759/cureus.9693

**Published:** 2020-08-12

**Authors:** Abdulraqeeb Taher, Adel A Alalwan, Naser Naser, Ola Alsegai, Ali Alaradi

**Affiliations:** 1 Nephrology, Salmaniya Medical Complex, Manama, BHR; 2 Pulmonary Medicine, Salmaniya Medical Complex, Manama, BHR; 3 Pathology and Laboratory Medicine, Salmaniya Medical Complex, Manama, BHR

**Keywords:** covid-19, acute kidney injury, covid-19 pneumonia, novel coronavirus

## Abstract

Background

Kidney disease accompanying coronavirus disease 2019 (COVID-19) is not well understood, and information about the presentation of acute kidney injury (AKI), its risk factors, and outcomes is scarce, particularly in Bahrain and the Gulf region. In this study, we aimed to determine the rate of AKI among patients hospitalized with COVID-19 pneumonia at a tertiary hospital in Bahrain and to describe the various aspects of AKI in these patients, including its relationship with respiratory failure and in-hospital mortality.

Methods

This retrospective observational cohort study of patients admitted with COVID-19 pneumonia from April 1 to May 31, 2020, excluded those aged less than 18 years, those with end-stage renal disease, and those with renal transplants. Clinical and laboratory patient data were collected. The Kidney Disease: Improving Global Outcomes (KDIGO) criteria were used to define AKI.

Results

The mean age across the 73 included patients was approximately 54 years; about 60% were men, and nearly 58% were Bahraini nationals. Of the patients, 39.7% (29) developed AKI during hospitalization, out of which 11.0% reached stage 1, 15.1% reached stage 2, and 13.7% reached stage 3. Of all patients, seven (9.6%) required hemodialysis. Chronic kidney disease conferred an increased risk for AKI (P = 0.003) as did critical COVID-19 status (P < 0.001) and the necessity for mechanical ventilation or intensive care admission (P < 0.001 for both). Additionally, AKI was significantly associated with a lower PaO_2_/FiO_2 _(partial pressure of arterial oxygen/percentage of inspired oxygen) ratio (P < 0.001) and a greater number of medications for COVID-19 pneumonia (P = 0.003). Finally, in-hospital death tolls were remarkably higher in patients with AKI (P < 0.001). No association was found between AKI and each of the following therapies: angiotensin-converting enzyme inhibitors or angiotensin receptor blockers, tocilizumab, and convalescent plasma.

Conclusions

The rate of AKI in patients hospitalized with COVID-19 pneumonia at our institution is relatively high and is strongly associated with disease severity, respiratory failure, and in-hospital mortality. Awareness of kidney disease in COVID-19 patients is crucial and of vital importance.

## Introduction

In December 2019, a novel coronavirus was recognized as the source of a series of cases of acute respiratory illness in Wuhan, a city in the Hubei Province of China. It spread rapidly, resulting in a global pandemic in which at least 15 million confirmed cases and more than 640,000 deaths were reported as of July 25, 2020 [1]. The World Health Organization named the disease coronavirus disease 2019 (COVID-19) and the culprit virus severe acute respiratory syndrome coronavirus 2 [2].

Bahrain identified its first case in late February 2020, and this was followed by an exponential growth in infections, resulting in more than 38,000 cases and 136 deaths as of July 25, 2020 [1,3]. As seen around the world, numerous hospitalizations, respiratory failures, and intensive care unit (ICU) admissions were seen [4,5].

While tending to patients with COVID-19, we found that the number of patients who developed acute kidney injury (AKI) was alarming: the rate was higher than that reported in China and was closer to the rate emerging from the USA [6,7]. To date, little has been published about AKI in COVID-19 in Bahrain and the Gulf region, and information about the presentation of AKI, its risk factors, and its outcomes is generally lacking [7].

In this study, we aimed to determine the rate of AKI among patients hospitalized with COVID-19 pneumonia at our institution and to describe the various aspects of the etiology of AKI in this patient population, including its relationship with respiratory failure and in-hospital mortality.

## Materials and methods

A retrospective observational cohort study was conducted at a government tertiary hospital, Salmaniya Medical Complex, in Bahrain. The medical records of 73 patients admitted with COVID-19 pneumonia from April 1 to May 31, 2020, were reviewed. Inclusion criteria were adult of age (≥18 years), patients showing positive polymerase chain reaction test for COVID-19 using a nasopharyngeal or sputum sample, and those diagnosed with COVID-19 pneumonia at admission. Exclusion criteria were patients with mild disease symptoms, a history of maintenance dialysis, and renal transplant recipients.

The study protocol and the waiver of written informed consent were approved by the secondary healthcare research sub-committee at the Ministry of Health in Bahrain. To maintain patient confidentiality and to ensure privacy, no personally identifying information was collected. However, clinical and laboratory data required for the study were gathered, which were sourced from the electronic health record system. The clinical data included age, sex, nationality, clinical presentation, contact and travel history, whether comorbidities were present, PaO_2_/FiO_2_ (partial pressure of arterial oxygen/percentage of inspired oxygen) ratio, disease severity, whether mechanical ventilation or ICU was required at admission, whether death occurred in hospital, whether AKI was present and its outcome, and the need for renal replacement therapy (RRT). Laboratory data were also collected at admission (complete blood count, renal function, electrolytes, disease-related inflammatory markers, and the estimated glomerular filtration rate [eGFR], which was calculated using the Cockcroft-Gault equation [8]) and during hospitalization, which included peak levels of creatinine and urea. All COVID-19 pneumonia treatments were recorded.

Disease severity was determined based on guidance from the Ministry of Health in Bahrain (published on April 15, 2020) [9] and the Chinese clinical guidance for COVID-19 pneumonia diagnosis and treatment (trial version 7) [10]. When no sign of pneumonia was seen on imaging, the case was considered to be mild. When fever, respiratory symptoms, and radiological signs of pneumonia were seen, the case was considered moderate. When dyspnea (respiratory rate ≥ 30 breaths/minute), hypoxia (oxygen saturation ≤ 93% at rest or PaO_2_/FiO_2_ ratio ≤ 300), or greater than 50% lung involvement was found on imaging within 24 to 48 hours of admission, the case was considered severe. Finally, when mechanical ventilation or ICU admission was required, the case was considered critical [9,10]. For this study, the lowest PaO_^2^_/FiO_2_ ratio logged during hospitalization was recorded.

The criteria identified by Kidney Disease: Improving Global Outcomes (KDIGO) were used to define AKI: stage 1 was an increase in serum creatinine by 26 μmol/L within 48 hours or by 1.5 to 1.9 times within seven days stage 2 was an increase by 2.9 times within seven days; and stage 3 was an increase by at least three times within seven days or initiation of RRT [11]. Patients were stratified according to the most severe AKI stage attained during the hospital stay.

Statistical Analysis

IBM SPSS Statistics 23 (IBM Corp., Armonk, NY, USA) was used for data entry and analysis. Frequencies and percentages were computed for categorical variables, whereas means and standard deviations were computed for continuous variables. Means and standard deviations of the continuous variables were computed relative to the categorical variables. Cross-tabulations were performed between two categorical variables. The Mann-Whitney test was used to determine whether the means of two groups were significantly different. The Kruskal-Wallis test was used to determine whether the means of more than two groups were significantly different. The chi-square test was used to determine whether two categorical variables were significantly related. The Fisher exact test was used to determine whether two categorical variables were significantly related in the case of small counts. In all statistical analyses, statistical significance was found when the P-value was less than 0.05.

## Results

The baseline characteristics of the 73 patients are shown in Table 1. Mean age was around 54 years, approximately 60% were men, and nearly 58% were Bahraini nationals. Many patients presented with fever (80.8%), cough (78.1%), and shortness of breath (49.3%), and some reported sore throat as well as gastrointestinal symptoms such as vomiting and diarrhea. A positive contact history was common (74%). Most (67.1%) patients had comorbidities, with diabetes and hypertension being the most common. Disease severity was most often moderate (49.3%), and the remainder were either severe (19.2%) or critical (31.5%). In total, 13 patients (17.8%) required mechanical ventilation; all of them died. Overall, 39.7% (29) developed AKI during hospitalization, out of which 11.0% reached stage 1, 15.1% reached stage 2, and 13.7% reached stage 3. Dialytic support was required for seven (9.6% of all patients), and the dialysis modality utilized was the prolonged intermittent RRT (also referred to as sustained low-efficiency dialysis [SLED]).

**Table 1:** Clinical characteristics of the study cohort (total = 73). PaO_2_, partial pressure of arterial oxygen; FiO_2_, percentage of inspired oxygen; ICU, intensive care unit; AKI, acute kidney injury; RRT, renal replacement therapy; SLED, sustained low-efficiency dialysis. Note: All variables are presented as n (%) for categorical variables or as mean ± SD for continuous variables.

Clinical characteristics			n (%)
1	Age (mean ± SD)		54.3 ± 13.5
2	Gender	Male	44 (60.3)
		Female	29 (39.7)
3	Nationality	Bahraini	42 (57.5)
		Non-Bahraini	31 (42.5)
4	Clinical presentation	Fever	59 (80.8)
		Cough	57 (78.1)
		Shortness of breath	36 (49.3)
		Sore throat	10 (13.7)
		Diarrhea	6 (8.2)
		Vomiting	2 (2.7)
		Loss of taste	1 (1.4)
		Hemoptysis	1 (1.4)
5	Contact history		54 (74)
6	Travel history		19 (26)
7	Comorbidities	Comorbidities	49 (67.1)
		Diabetes mellitus	33 (45.2)
		Hypertension	31 (42.5)
		Cardiovascular disease	9 (12.3)
		Chronic kidney disease	6 (8.2)
		Obesity	3 (4.1)
		Malignancy	5 (6.8)
		Others	8 (11)
8	PaO_2_/FiO_2_ ratio (mean ± SD)		276.9 ± 147
9	Disease severity	Moderate disease	36 (49.3)
		Severe disease	14 (19.2)
		Critical disease	23 (31.5)
10	Mechanical ventilation		13 (17.8)
11	ICU admission		23 (31.5)
12	In-hospital death		13 (17.8)
13	AKI		29 (39.7)
14	AKI stage	Stage 1	8 (11)
		Stage 2	11 (15.1)
		Stage 3	10 (13.7)
15	Treatment of AKI	No RRT (conservative treatment)	22 (30.1)
		RRT (SLED)	7 (9.6)
16	Outcomes of AKI	Recovery	16 (21.9)
		Dialysis dependent	1 (1.4)
		Death	12 (16.4)

Table 2 shows the laboratory data. Mean values for C-reactive protein, the erythrocyte sedimentation rate, D-dimer, lactate dehydrogenase, ferritin, and creatine kinase were elevated. On the other hand, the mean absolute lymphocyte count was in the low normal range, driven by those (approximately 36% of the patients) with lymphopenia (absolute lymphocyte count < 1000 cells/μL). Most had normal hemoglobin values at admission; however, 26% had thrombocytopenia (platelets < 150 × 10^9^/L). At admission, procalcitonin levels were primarily normal, as were those for alanine aminotransferase, urea, creatinine, eGFR, and electrolytes.

**Table 2:** Laboratory parameters of the study cohort with reference ranges (total = 73). WBC, white blood cells; INR, international normalized ratio; CRP, C-reactive protein; PCT, procalcitonin; ESR, erythrocyte sedimentation rate; LDH, lactate dehydrogenase; CK, creatine kinase; ALT, alanine aminotransferase; eGFR, estimated glomerular filtration rate. *Indicates that the laboratory parameter (absolute lymphocyte count, platelet count) has additional descriptive analysis. Note: All laboratory values were collected at admission, except for the peak values of creatinine and urea, which were recorded during hospitalization. All variables are presented as n (%) for categorical variables or as mean ± SD for continuous variables.

Laboratory parameter		Mean ± SD	Reference range
1	WBC	6.56 ± 2.53 × 10^9^	3.6–9.6 × 10^9^/L
2	Lymphocyte %	24.52 ± 13.07	20.5–55.1%
3	Absolute lymphocyte count*	1590.77 ± 1179.02	≥ 1000 cells/μL
4	Hemoglobin	13.41 ± 1.95	12–14.5 g/dL
5	Platelets*	227.26 ± 118.15 × 10^9^	150–400 × 10^9^/L
6	INR	1.04 ± 0.1	0.61–1.17
7	CRP	89.33 ± 73.01	0–3 mg/L
8	PCT	0.41 ± 1.09	0–0.5 μg/L
9	ESR	67.93 ± 36.39	0–29 mm/hour
10	D-dimer	2.78 ± 4.31	0.09–0.33 mg/L
11	LDH	357.29 ± 219.14	135–225 U/L
12	Ferritin	946.01 ± 647.12	16–323 μg/L
13	CK	169.62 ± 223.41	35–232 U/L
14	ALT	38.36 ± 18.17	<41 U/L
15	eGFR	93.36 ± 32.11	>90 mL/min
16	Baseline urea	5.99 ± 3.63	3.2–8.2 mmol/L
17	Baseline creatinine	72.16 ± 33.59	53–97 μmol/L
18	Peak urea	11.33 ± 10.86	3.2–8.2 mmol/L
19	Peak creatinine	134.86 ± 137.94	53–97 μmol/L
20	Sodium	137.7 ± 5.15	132–146 mmol/L
21	Potassium	4.26 ± 0.6	3.5–5.5 mmol/L
22	Bicarbonate	25.55 ± 3.37	24–32 mmol/L

*Descriptive analysis of absolute lymphocyte count and platelet count (total = 73)			
Laboratory parameter			n (%)
1	Absolute lymphocyte count	<1000 cells/μL	26 (35.6)
		≥1000 cells/μL	47 (64.4)
2	Platelet count	<150 × 10^9^/L	19 (26)
		≥150 × 10^9^/L	54 (74)

The primary treatments for COVID-19 pneumonia are shown in Table 3. In most cases, the patient was initiated on hydroxychloroquine, empiric antibiotic therapy, and antiviral therapy according to guidance from the Ministry of Health in Bahrain (published April 15, 2020) [9]. Tocilizumab, a monoclonal antibody to the interleukin-6 receptor, and convalescent plasma were used in 16.4% and 26.0%, respectively. Heparin or low-molecular-weight heparin was widely administered. In contrast, the use of steroids was limited (11% of all patients).

**Table 3:** COVID-19 treatments used during hospitalization. LMWH, low-molecular-weight heparin

Treatment			n (%)
1	Hydroxychloroquine		71 (97.3)
2	Antivirals	Ribavirin	27 (37)
		Lopinavir-ritonavir	67 (91.8)
		Interferon	20 (27.4)
		Oseltamivir	27 (37)
3	Antibiotics	Azithromycin	61 (83.6)
		Doxycycline	6 (8.2)
		Ceftriaxone	19 (26)
		Augmentin	5 (6.8)
		Piperacillin-tazobactam	36 (49.3)
		Meropenem	12 (16.4)
		Linezolid	12 (16.4)
		Levofloxacin	1 (1.4)
		Vancomycin	1 (1.4)
4	Tocilizumab		12 (16.4)
5	Convalescent plasma therapy		19 (26)
6	Heparin or LMWH		64 (87.7)
7	Steroids		8 (11)
8	Number of COVID-19 medications	3–4	30 (41.1)
		5–6	32 (43.8)
		7–8	11 (15.1)

**Figure 1:**
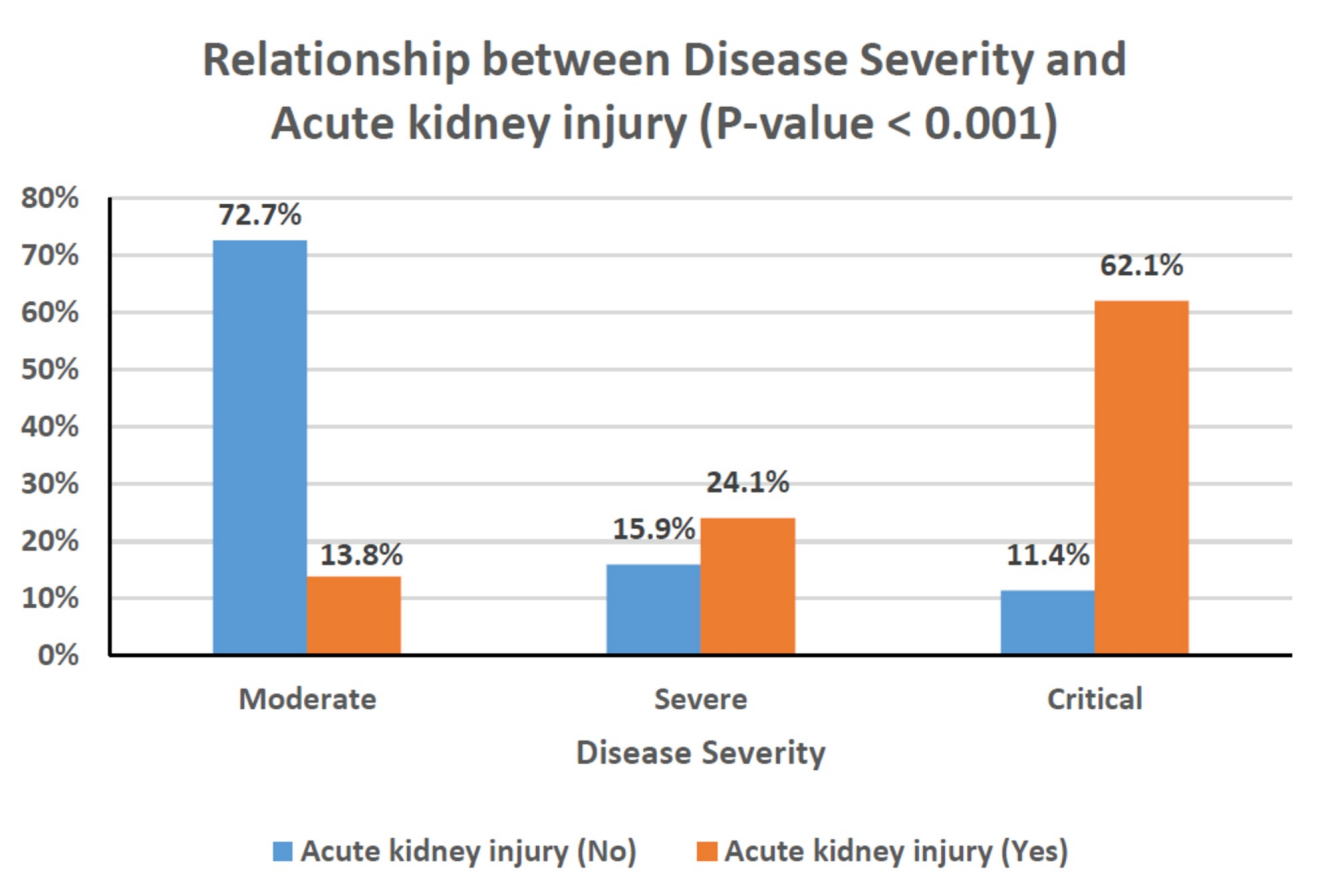
Clustered bar chart of the relationship between disease severity and acute kidney injury (P < 0.001).

**Figure 2:**
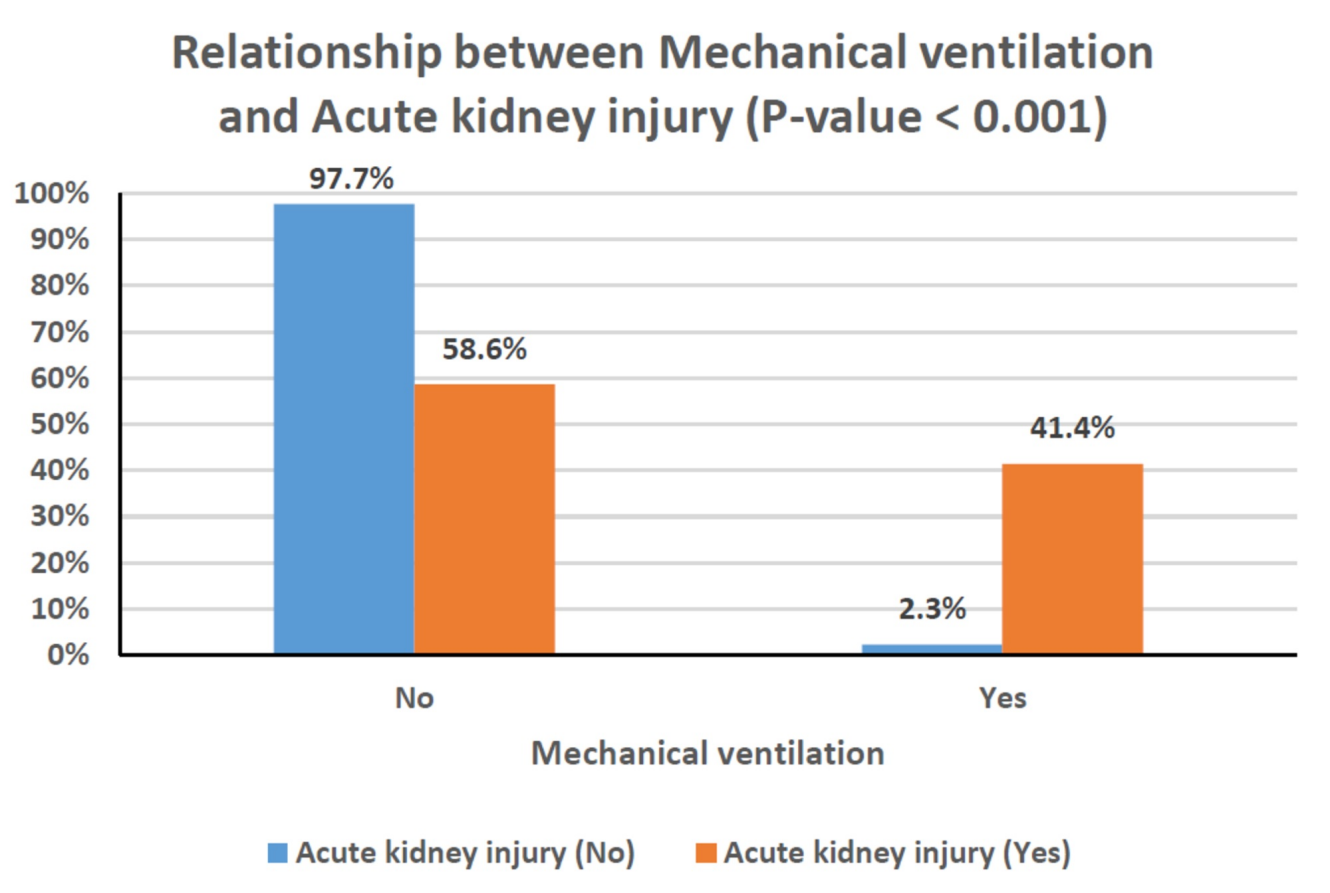
Clustered bar chart of the relationship between mechanical ventilation and acute kidney injury (P < 0.001).

**Figure 3:**
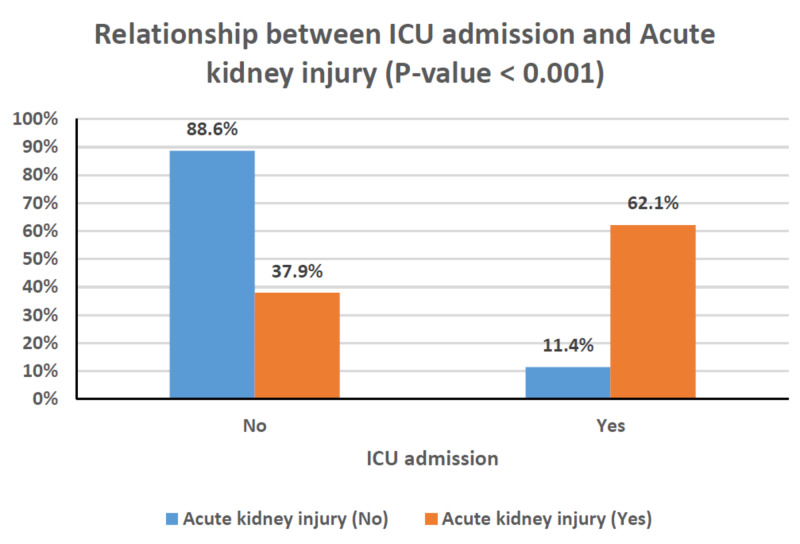
Clustered bar chart of the relationship between ICU admission and acute kidney injury (P < 0.001). ICU, intensive care unit

**Figure 4:**
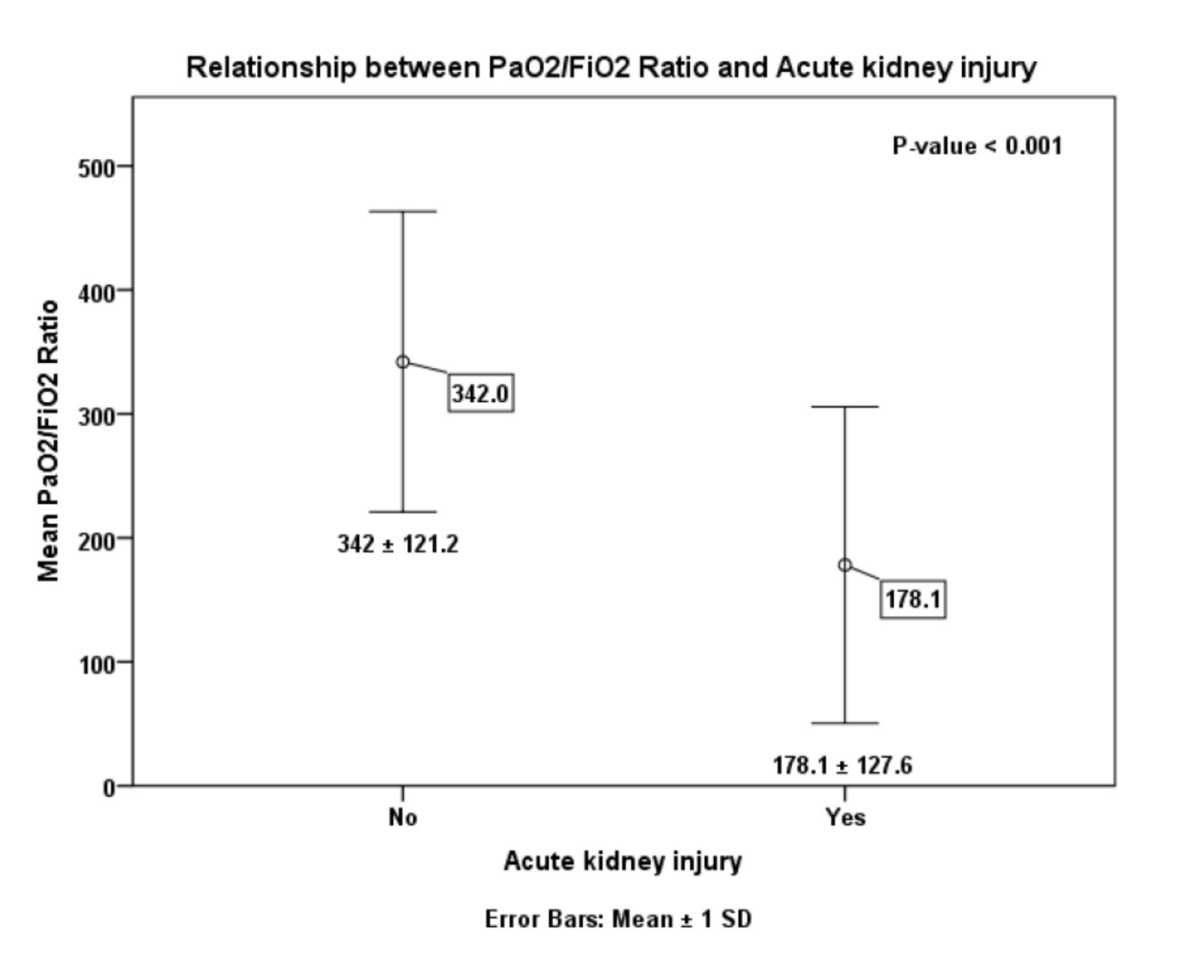
Error bars chart of the relationship between the PaO2/FiO2 ratio and acute kidney injury (P < 0.001). PaO_2_, partial pressure of arterial oxygen; FiO_2_, percentage of inspired oxygen.

**Figure 5:**
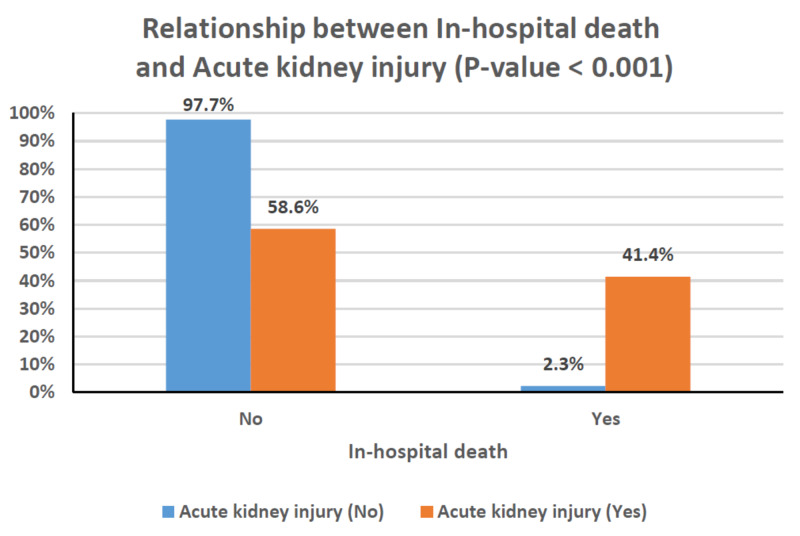
Clustered bar chart of the relationship between in-hospital death and acute kidney injury (P < 0.001).

Table 4 shows the relationships between clinical characteristics and the development of AKI. Older age, male gender, and the presence of comorbidities occurred more frequently among patients with AKI; however, these associations did not reach statistical significance. Patients with chronic kidney disease, in particular, had a greater risk of developing AKI (P = 0.003). The rates of AKI were also significantly higher in patients with critical disease (P < 0.001), and, as a result, they were substantially greater in those requiring mechanical ventilation or ICU admission (P < 0.001 for both; Figures 1-3). This can also be observed in the PaO_2_/FiO_2_ ratio, where lower values were significantly associated with the presence of AKI (P < 0.001; Figure 4). In-hospital deaths occurred more frequently among patients with AKI (P < 0.001; Figure 5). Notably, stage 3 AKI was significantly associated with higher rates of mechanical ventilation and in-hospital death (P = 0.007).

**Table 4:** Clinical characteristics of the study cohort stratified by AKI status and the stages of AKI. AKI, acute kidney injury; PaO_2_, partial pressure of arterial oxygen; FiO_2_, percentage of inspired oxygen; ICU, intensive care unit Note: All variables are presented as n (%) for categorical variables or as mean ± SD for continuous variables. In all statistical analyses, a P-value of less than 0.05 was considered statistically significant.

Variables			AKI		P-value	AKI stage			P-value
			No (n = 44)	Yes (n = 29)		Stage 1 (n = 8)	Stage 2 (n = 11)	Stage 3 (n = 10)	
1	Age		52.2 ± 13.1	57.5 ± 13.7	0.104	50.4 ± 11.5	61.7 ± 16.3	58.6 ± 10.8	0.217
2	Gender								
	a	Male	23 (52.3)	21 (72.4)	0.085	8 (100)	7 (63.6)	6 (60)	0.120
	b	Female	21 (47.7)	8 (27.6)		0 (0)	4 (36.4)	4 (40)	
3	All comorbidities		26 (59.1)	23 (79.3)	0.072	6 (75)	9 (81.8)	8 (80)	0.934
4	Comorbidities								
	a	Diabetes mellitus	16 (36.4)	17 (58.6)	0.062	4 (50)	6 (54.5)	7 (70)	0.652
	b	Hypertension	16 (36.4)	15 (51.7)	0.194	3 (37.5)	4 (36.4)	8 (80)	0.087
	c	Cardiovascular disease	3 (6.8)	6 (20.7)	0.142	2 (25)	1 (9.1)	3 (30)	0.468
	d	Chronic kidney disease	0 (0)	6 (20.7)	0.003	2 (25)	0 (0)	4 (40)	0.073
	e	Obesity	1 (2.3)	2 (6.9)	0.559	1 (12.5)	1 (9.1)	0 (0)	0.545
	f	Malignancy	2 (4.5)	3 (10.3)	0.380	0 (0)	3 (27.3)	0 (0)	0.065
	g	Others	2 (4.5)	6 (20.7)	0.052	3 (37.5)	3 (27.3)	0 (0)	0.118
5	PAO_2_/FIO_2_ ratio		342 ± 121.2	178.1 ± 127.6	<0.001	261.9 ± 160.7	180.1 ± 120.3	108.9 ± 54.2	0.076
6	Disease severity								
	a	Moderate	32 (72.7)	4 (13.8)	<0.001	3 (37.5)	1 (9.1)	0 (0)	0.076
	b	Severe	7 (15.9)	7 (24.1)		2 (25)	4 (36.4)	1 (10)	
	c	Critical	5 (11.4)	18 (62.1)		3 (37.5)	6 (54.5)	9 (90)	
7	Mechanical ventilation		1 (2.3)	12 (41.4)	<0.001	1 (12.5)	3 (27.3)	8 (80)	0.007
8	ICU admission		5 (11.4)	18 (62.1)	<0.001	3 (37.5)	6 (54.5)	9 (90)	0.060
9	In-hospital death		1 (2.3)	12 (41.4)	<0.001	1 (12.5)	3 (27.3)	8 (80)	0.007

As shown in Table 5, no significant relationships were found between the development of AKI and the baseline values for creatinine, urea, and eGFR. However, as expected, the peak values for creatinine and urea were remarkably higher in patients with AKI (P < 0.001 for both). In addition, proteinuria and hematuria were more common among patients with AKI, reaching around 83% and 38%, respectively. Their presence also corresponded with a significantly greater risk of AKI. The effects of specific treatments on the rate of AKI are shown in Table 5. No considerable association was found between AKI and each of the following: angiotensin-converting enzyme inhibitors (ACE-Is) or angiotensin receptor blockers (ARBs), tocilizumab, and convalescent plasma therapy. Nevertheless, a significant association was noted between the number of medications used for COVID-19 pneumonia and the development of AKI (P = 0.003).

**Table 5:** Parameters of kidney function and selected treatments stratified by AKI status and the stages of AKI. AKI, acute kidney injury; eGFR, estimated glomerular filtration rate; ACE-Is, angiotensin-converting enzyme inhibitors; ARBs, angiotensin receptor blockers Note: All variables were presented in the form of n (%) for categorical variables or mean ± SD for continuous variables. In all statistical analyses, a P-value of less than 0.05 was considered statistically significant.

Variables			AKI		P-value	AKI stage			P-value
			No (n = 44)	Yes (n = 29)		Stage 1 (n = 8)	Stage 2 (n = 11)	Stage 3 (n = 10)	
Parameters of kidney function									
1	Baseline urea		5.1 ± 1.5	7.4 ± 5.2	0.054	6.3 ± 3.2	4.9 ± 1.5	11 ± 7.1	0.029
2	Baseline creatinine		64.6 ± 14.9	83.7 ± 48.3	0.349	82.6 ± 29.6	56.6 ± 10	114.5 ± 67.2	0.008
3	Peak urea		5.6 ± 1.6	20 ± 13	<0.001	12.9 ± 5.7	12.4 ± 7.4	34.2 ± 10	<0.001
4	Peak creatinine		67 ± 15	237.9 ± 174.2	<0.001	131.7 ± 37.2	129.2 ± 24	442.3 ± 147.7	<0.001
5	eGFR		98.5 ± 28.7	85.6 ± 35.8	0.182	91.6 ± 39.4	99.9 ± 29.2	65.1 ± 33.1	0.114
6	Proteinuria								
	a	Negative	30 (68.2)	5 (17.2)	<0.001	3 (37.5)	1 (9.1)	1 (10)	0.222
	b	1+	13 (29.5)	10 (34.5)		1 (12.5)	6 (54.5)	3 (30)	
	c	2+ to 3+	1 (2.3)	14 (48.3)		4 (50)	4 (36.4)	6 (60)	
7	Hematuria:								
	a	Negative	40 (90.9)	18 (62.1)	0.004	8 (100)	6 (54.5)	4 (40)	0.117
	b	1+	4 (9.1)	6 (20.7)		0 (0)	3 (27.3)	3 (30)	
	c	2+ to 3+	0 (0)	5 (17.2)		0 (0)	2 (18.2)	3 (30)	
Selected treatments									
1	ACE-Is or ARBs		13 (29.5)	6 (20.7)	0.399	1 (12.5)	1 (9.1)	4 (40)	0.174
2	Tocilizumab		5 (11.4)	7 (24.1)	0.200	0 (0)	2 (18.2)	5 (50)	0.041
3	Convalescent plasma therapy		11 (25)	8 (27.6)	0.805	2 (25)	4 (36.4)	2 (20)	0.691
Number of COVID-19 medications									
4	a	3–4	25 (56.8)	5 (17.2)	0.003	3 (37.5)	1 (9.1)	1 (10)	0.031
	b	5–6	14 (31.8)	18 (62.1)		5 (62.5)	9 (81.8)	4 (40)	
	c	7–8	5 (11.4)	6 (20.7)		0 (0)	1 (9.1)	5 (50)	

## Discussion

The most frequent serious manifestation of COVID-19 is pneumonia, characterized primarily by fever, cough, dyspnea, and bilateral infiltrates on chest images [4]. Nevertheless, other features, including upper respiratory tract symptoms, vomiting, diarrhea, myalgias, and smell or taste disorders, can be present [12]. Adults of advanced age or with underlying medical comorbidities frequently develop severe disease [13,14]. Diabetes, hypertension, cardiovascular disease, chronic kidney disease, obesity, chronic lung disease, and malignancy are among the most common risk factors [13]. In addition, particular laboratory features have been associated with worse disease outcomes including lymphopenia and elevations in the levels of lactate dehydrogenase, D-dimer, creatine kinase, troponin, and inflammatory markers (C-reactive protein, ferritin, etc.) [15]. Additionally, AKI has been proposed as a marker of disease severity [7].

Patients with COVID-19 can present with AKI as part of their illness. The reported incidence ranges from 3% to 37% based on observational data from China and the USA [6,7,16]. Of our study population, 39.7% developed AKI during hospitalization, a rate greater than that observed in China and other areas, but somewhat consistent with anecdotal reports emerging from the USA [17]. Cheng et al. noted a rate of 5.1% (37 of 701) in Wuhan, China [6], but Hirsch et al. reported 36.6% of 5,449 patients in New York [7]. The difference can be partially explained by variations in disease severity. In particular, mechanical ventilation was required by 13.4% of the cohort studied by Cheng et al. but 21.8% of those studied by Hirsch et al.; in our patient population, that segment was 17.8% of the cohort. Overall, AKI incidence in COVID-19 appears to vary by geographical location and the proportion of critically ill patients in each study population.

Kidney disease in COVID-19 patients has several etiologies that have not yet been fully elucidated, and it can manifest as AKI, hematuria, or proteinuria [6,7,18]. Although AKI might be attributable to hypotension and decreased kidney perfusion secondary to hemodynamic factors or associated sepsis, direct viral infection of the kidneys also plays a role [19]. Invasion of the culprit virus is mediated primarily by binding to the angiotensin-converting enzyme 2 protein, which is expressed in many tissues, including lungs, heart, and kidneys. Renal tubular and podocyte damage have been linked to virus-mediated injury as well as COVID-19-associated macrophage activation and a cytokine storm, which result in hypercoagulation and microangiopathy [20]. In addition, organ crosstalk between the injured lung, the heart, and the kidney can worsen the pathology of AKI [18,20].

In our cohort, the most evident risk factors for AKI development were also indicators of severe COVID-19, specifically the PaO_2_/FiO_2_ ratio, the need for ventilator support, and ICU admission (Table 4) [15]. The reported risk factors for poor outcomes of COVID-19, such as increased age, male sex, and comorbidities, were also more prevalent in patients with AKI (Table 4) [14]. Moreover, the rate of AKI was found to increase as the number of medications for treating COVID-19 pneumonia increased (Table 5). This is likely because some of the medications are nephrotoxic (ribavirin, piperacillin-tazobactam, meropenem, etc.), and combining them could contribute to AKI pathology [21]. Interest in the roles of ACE-Is and ARBs in COVID-19 has been notable [22,23]. In our study population, the use of these drugs at hospital admission was not related to AKI risk: 20.7% of patients with AKI used ACE-Is or ARBs compared to 29.5% of those without AKI (Table 5).

AKI occurs in temporal association with respiratory failure in severe COVID-19 disease and is associated with a poor prognosis [7]. Our findings support a significant association between AKI and COVID-19 disease severity and, in turn, the rates of mechanical ventilation and ICU admission (Figures 1-3). Among patients with AKI, 41.4% required mechanical ventilation, of whom 80% were in stage 3 AKI (Table 4). In cases where invasive mechanical ventilation is necessary, biotrauma, barotrauma, the release of inflammatory mediators, and hemodynamic compromise can occur. These mechanisms can further contribute to kidney injury, eventually leading to an impaired glomerular filtration rate or even renal failure [24]. Moreover, the associations between AKI and both COVID-19 disease severity and respiratory failure reflect on the rate of in-hospital mortality as well (Figure 5). Our results agree with those from around the world, particularly the USA and China, which point to dire prognoses for COVID-19 patients with AKI, and even bleaker prognoses for those requiring RRT [6,7]. Of the 29 patients with AKI in our study, 12 died (Table 4), and of those requiring dialytic support, one completely recovered, one became dialysis-dependent, and five died (Table 1).

The optimal approach to COVID-19 treatment remains uncertain. The Ministry of Health in Bahrain constantly updates the guidelines for treating COVID-19 pneumonia to keep pace with emerging clinical data from around the world [9,25]. Several therapies administered to our study population have since been discontinued and replaced with more effective medications with more favorable side-effect profiles [25]. Most notable is the inclusion of steroid therapy, using dexamethasone as the standard when supplemental oxygen is required, as suggested by the RECOVERY trial [26]. The use of systemic steroids was initially restricted due to concerns regarding their effects on delaying viral clearance, as noted in previous coronavirus outbreaks [27]. Triple antiviral therapy (ribavirin, lopinavir-ritonavir, and interferon) was also discontinued and replaced with either favipiravir or remdesivir. Additionally, oseltamivir and hydroxychloroquine are largely avoided [25]. However, the recommendations for tocilizumab and convalescent plasma therapy in severe COVID-19 pneumonia remain unchanged [9,25]. Our results showed that neither seemed to significantly affect the rate of AKI. An explanation might be that these are therapies of last resort in severe and critical cases wherein the pathology of AKI is ongoing and difficult to reverse [20,28]. More extensive observational studies and controlled trials are needed to find associations between each of these treatments and the development of AKI in COVID-19.

In our cohort, most patients with AKI recovered with conservative care alone; only seven needed RRT. Although continuous RRT (CRRT) is the preferred dialysis modality for critically ill patients with AKI, SLED was used at our facility [29]. As with CRRT, SLED allows for improved hemodynamic stability through gradual solute and volume removal [30]. Amid the COVID-19 pandemic, the use of SLED is recommended when CRRT machines are insufficient and equipment cannot meet the growing demands. The shorter sessions in SLED (6 to 12 hours) compared to CRRT (≥24 hours) facilitate machine and staffing availabilities [29].

Limitations

This study has limitations. First, causal relationships between the different variables and AKI cannot be confirmed given the observational nature of this study. Second, the electronic health information records were the sole source of data, including the identification of AKI. Third, these findings cannot be applied to outpatient AKI settings because non-hospitalized patients were not part of the cohort. Finally, these results cannot be generalized to the entire population with COVID-19 pneumonia in Bahrain because of the relatively small sample size and the single-centered nature of the study. Multi-center studies could confirm the rate of AKI in COVID-19 pneumonia.

## Conclusions

This single-center study shows that AKI is a relatively common finding among patients hospitalized with COVID-19 pneumonia in Bahrain and that it is strongly linked to disease severity, respiratory failure, and the need for mechanical ventilation as well as ICU admission. The development of AKI in patients hospitalized with COVID-19 pneumonia signifies poor prognosis. Awareness and early detection of kidney involvement in these patients, accompanied by effective management, could reduce the morbidity and mortality rates of COVID-19. Furthermore, a better understanding of the causes of AKI and patient outcomes necessitates broader studies.

## References

[1] (2020). COVID-19 Dashboard by the Center for Systems Science and Engineering at Johns Hopkins University. https://gisanddata.maps.arcgis.com/apps/opsdashboard/index.html#/bda7594740fd40299423467b48e9ecf6.

[2] (2020). WHO Director-General's remarks at the media briefing on 2019-nCoV on 11 February 2020. https://www.who.int/dg/speeches/detail/who-director-general-s-remarks-at-the-media-briefing-on-2019-ncov-on-11-february-2020.

[3] (2020). Bahrain coronavirus case no. 1: a school bus driver. https://gulfnews.com/world/gulf/bahrain/bahrain-coronavirus-case-no-1-a-school-bus-driver-1.1582613552064.

[4] Wang D, Hu B, Hu C (2020). Clinical characteristics of 138 hospitalized patients with 2019 novel coronavirus-infected pneumonia in Wuhan, China. JAMA.

[5] Onder G, Rezza G, Brusaferro S (2020). Case-fatality rate and characteristics of patients dying in relation to COVID-19 in Italy. JAMA.

[6] Cheng Y, Luo R, Wang K (2020). Kidney disease is associated with in-hospital death of patients with COVID-19. Kidney Int.

[7] Hirsch JS, Ng JH, Ross DW (2020). Acute kidney injury in patients hospitalized with COVID-19. Kidney Int.

[8] Cockcroft DW, Gault MH (1976). Prediction of creatinine clearance from serum creatinine. Nephron.

[9] (2020). Bahrain COVID-19 protocols (Published April 15, 2020). https://www.nhra.bh/Media/Announcement/MediaHandler/GenericHandler/documents/Announcements/NHRA_News_MOHALERT_BahrainCOVID-19Protocols_20200417.pdf.

[10] (2020). Diagnosis and treatment protocol for novel coronavirus pneumonia (Trial Version 7). http://www.kankyokansen.org/uploads/uploads/files/jsipc/protocol_V7.pdf.

[11] Khwaja A (2012). KDIGO clinical practice guidelines for acute kidney injury. Nephron Clin Pract.

[12] Guan W, Ni Z, Hu Y (2020). Clinical characteristics of coronavirus disease 2019 in China. N Engl J Med.

[13] Wu Z, McGoogan JM (2020). Characteristics of and important lessons from the coronavirus disease 2019 (COVID-19) outbreak in China: summary of a report of 72 314 cases from the Chinese Center for Disease Control and Prevention. JAMA.

[14] Petrilli CM, Jones SA, Yang J (2020). Factors associated with hospital admission and critical illness among 5279 people with coronavirus disease 2019 in New York City: prospective cohort study. BMJ.

[15] Wu C, Chen X, Cai Y (2020). Risk factors associated with acute respiratory distress syndrome and death in patients with coronavirus disease 2019 pneumonia in Wuhan, China. JAMA Intern Med.

[16] Ng JJ, Luo Y, Phua K, Choong AMTL (2020). Acute kidney injury in hospitalized patients with coronavirus disease 2019 (COVID- 19): a meta-analysis [Online ahead of print]. J Infect.

[17] Abelson R, Fink S, Nicholas K, Thomas K (2020). An Overlooked, Possibly Fatal Coronavirus Crisis: A Dire Need for Kidney Dialysis. New York Times.

[18] Su H, Yang M, Wan C (2020). Renal histopathological analysis of 26 postmortem findings of patients with COVID-19 in China. Kidney Int.

[19] Delvaeye M, Conway EM (2009). Coagulation and innate immune responses: can we view them separately?. Blood.

[20] Batlle D, Soler MJ, Sparks MA (2020). Acute kidney injury in COVID- 19: emerging evidence of a distinct pathophysiology. J Am Soc Nephrol.

[21] Schetz M, Dasta J, Goldstein S, Golper T (2005). Drug-induced acute kidney injury. Curr Opin Crit Care.

[22] Vaduganathan M, Vardeny O, Michel T, McMurray JJV, Pfeffer MA, Solomon SD (2020). Renin-angiotensin-aldosterone system inhibitors in patients with Covid-19. N Engl J Med.

[23] Li J, Wang X, Chen J, Zhang H, Deng A (2020). Association of renin-angiotensin system inhibitors with severity or risk of death in patients with hypertension hospitalized for coronavirus disease 2019 (COVID-19) infection in Wuhan, China. JAMA Cardiol.

[24] Joannidis M, Forni LG, Klein SJ (2020). Lung-kidney interactions in critically ill patients: consensus report of the Acute Disease Quality Initiative (ADQI) 21 Workgroup. Intensive Care Med.

[25] Bahrain COVID-19 protocols (July 1, 2020 2020 (2020). Bahrain COVID-19 protocols (Published July 1, 2020). https://www.nhra.bh/Media/Announcement/MediaHandler/GenericHandler/documents/Announcements/NHRA_News_MOHALERT_BahrainCOVID-19NationalProtocols_20200701.pdf.

[26] RECOVERY Collaborative Group, Horby P, Lim WS (2020). Dexamethasone in hospitalized patients with Covid-19 - preliminary report [Online ahead of print]. N Engl J Med.

[27] Hui DS (2018). Systemic corticosteroid therapy may delay viral clearance in patients with Middle East respiratory syndrome coronavirus infection. Am J Respir Crit Care Med.

[28] ElSeirafi MM, Hasan HM, Sridharan K, Zamoori A, Alkhawaja S, Pasha SAA (2020). Efficacy and safety of tocilizumab in critically ill adults with COVID-19 infection in Bahrain: a report of 5 cases. Respir Med Case Rep.

[29] (2020). Acute Kidney Injury and Renal Replacement Therapy. https://www.covid19treatmentguidelines.nih.gov/critical-care/acute-kidney-injury/.

[30] Edrees F, Li T, Vijayan A (2016). Prolonged intermittent renal replacement therapy. Adv Chronic Kidney Dis.

